# A Novel Nuclear Function for the Interleukin-17 Signaling Adaptor Protein Act1

**DOI:** 10.1371/journal.pone.0163323

**Published:** 2016-10-10

**Authors:** Sharlene Velichko, Xu Zhou, Lingxiang Zhu, Johnathon David Anderson, Reen Wu, Yin Chen

**Affiliations:** 1 The Center for Comparative Respiratory Biology and Medicine, Department of Internal Medicine, University of California Davis, Davis, California, 95616, United States of America; 2 Department of Pharmacology and Toxicology, School of Pharmacy, University of Arizona, Tucson, Arizona, 85721, United States of America; National Jewish Health, UNITED STATES

## Abstract

In the context of the human airway, interleukin-17A (IL-17A) signaling is associated with severe inflammation, as well as protection against pathogenic infection, particularly at mucosal surfaces such as the airway. The intracellular molecule Act1 has been demonstrated to be an essential mediator of IL-17A signaling. In the cytoplasm, it serves as an adaptor protein, binding to both the intracellular domain of the IL-17 receptor as well as members of the canonical nuclear factor kappa B (NF-κB) pathway. It also has enzymatic activity, and serves as an E3 ubiquitin ligase. In the context of airway epithelial cells, we demonstrate for the first time that Act1 is also present in the nucleus, especially after IL-17A stimulation. Ectopic Act1 expression can also increase the nuclear localization of Act1. Act1 can up-regulate the expression and promoter activity of a subset of IL-17A target genes in the absence of IL-17A signaling in a manner that is dependent on its N- and C-terminal domains, but is NF-κB independent. Finally, we show that nuclear Act1 can bind to both distal and proximal promoter regions of *DEFB4*, one of the IL-17A responsive genes. This transcriptional regulatory activity represents a novel function for Act1. Taken together, this is the first report to describe a non-adaptor function of Act1 by directly binding to the promoter region of IL-17A responsive genes and directly regulate their transcription.

## Introduction

The airway epithelium plays a critical role in host immunity. Not only does it act as a physical barrier to prevent the passage of opportunistic pathogens into the underlying tissue, it also orchestrates both an innate and adaptive immune response in lung tissue by secreting various chemokines, cytokines and antimicrobial proteins. Recent literature has shown that IL-17A can stimulate airway epithelial cells to express mucins as well as various neutrophil-recruiting chemokines, cytokines and antimicrobial proteins [1–6]. IL-17A plays a critically important role in mucosal surface resistance to infection by both extracellular and intracellular bacterial pathogens, as animals deficient in either IL-17A or its receptor, IL-17RA, demonstrate increased susceptibility to bacterial infection [6–11]. While necessary for host defense, uncontrolled IL-17A signaling may lead to inflammation. Increased IL-17A levels in the airway have been associated with chronic inflammatory diseases such as COPD and severe asthma, hallmarks of which include airway remodeling and neutrophilia [12–15]. Additionally, exogenous administration of IL-17A into mouse airways was shown to cause neutrophilia and airway remodeling [3].

Act1 (also known as CIKS) is a major downstream modulator of both interleukin-17 (IL-17)A/IL-17F and IL-25 (IL-17E) signaling, as demonstrated by the fact that Act1-null mice are largely unresponsive to these cytokines [16–18].

Act1 was first cloned in the year of 2000 by two separate groups, and was shown to be able to activate both the c-June N-terminal kinase (JNK) and canonical nuclear factor kappa B (NF-κB) pathways [19, 20]. Act1 and all known IL-17 receptor family members were shown to share a conserved domain with distant homology to the TIR domain of Toll-like receptors [21]. This domain was termed the SEFIR domain and gave the first indication that Act1 might be involved in the signaling function of the newly identified IL-17 cytokine family. It was subsequently shown that Act1 could bind to several IL-17 receptor family members, via homotypic interactions between their respective SEFIR domains [16, 22]. The receptor subunits for IL-17A (i.e. IL-17RA and IL-17RC) and the receptor subunit for IL-25 (i.e. IL-17RB) have been shown to co-immunoprecipitate with Act1 in mammalian cells [16, 17, 22–24]. Due to its ability to interact with IL-17 receptors, as well as with downstream mediators of the NF-κB pathway, including IKK-α/IKK-β, NEMO/IKK-γ, TAK1 and TRAF6, Act1 is thought to function as an adaptor molecule, linking the receptors to the NF-κB pathway [20, 25, 26]. However, different from other adaptor molecules, Act1 has been recently reported to function as an E3 ubiquitin ligase. TRAF6 was identified as being one of its downstream targets. This enzymatic activity was found to be necessary for full IL-17A mediated NF-κB and JNK activation [27]. Additionally, other IL-17A mediated signaling functions, such as mRNA stabilization and ERK phosphorylation, are Act1 dependent but TRAF6 independent, indicating that there are other signaling pathways downstream of Act1 as well [22, 28, 29].

In this report, we describe a novel mechanism by which Act1 directly affects gene expression by acting as a nuclear transcriptional activator, resulting in the up-regulation of a subset of IL-17A responsive genes, including ones identified in our previous studies, including: beta-defensin 4 (DEFB4), IL-19 and CCL20 [4, 5, 30]. This may represent a novel pathway by which IL-17A signals.

## Materials and Methods

### 1. Cell lines, Antibodies and Reagents

Human bronchial tissues were purchased from the commercial source (National Disease Research Interchange) as described before [31, 32]. No tissues from patients diagnosed with lung-related diseases were used. All those lungs were either autopsy leftovers or were rejected for transplant. They were sent to us with arbitrary numerical code. No identity link to the actual patient can be identified. NDRI is a not-for-profit commercial exchanged HBE1, a human papillomavirus immortalized cell line, was obtained and maintained as described previously [33]. A549 cells were obtained from the American Type Culture Collection and maintained in RPMI + 10% fetal bovine serum. Human IL-17A was purchased from R&D Systems. Anti-FLAG (M2) and anti-β actin mouse monoclonal antibodies were purchased from Sigma-Aldrich. Rabbit anti-Act1 antibody was purchased from Santa Cruz Biotechnology (Santa Cruz, CA). Anti-nucleolin mouse monoclonal antibody was purchased from Research Diagnostics Inc.. Rabbit anti-myc antibody was purchased from Abcam. Horseradish peroxidase (HRP) conjugated secondary antibodies and Clean-Blot HRP for western blot were purchased from Thermo Fisher Scientific. Alexa-488 conjugated secondary antibodies for indirect immunofluorescence were purchased from Invitrogen. Non-targeting (NT) siRNA was purchased from Ambion and Act1 siRNA was purchased from Sigma-Aldrich. DEFB4 ELISA construction kit was purchased from Antigenix America and performed according to the manufacturer’s suggested protocol. NF-κB Activation Inhibitor III was purchased from EMD Biosciences.

### 2. Plasmids and Transfection

The DEFB4-wt-LUC, DEFB4-mut-LUC and CCL20-LUC luciferase reporter constructs were described previously [5, 34]. The DEFB4-wt-LUC construct was used as a template to create the 1.0kb, 1.4kb and 1.8kb constructs by PCR amplification to add KpnI and HindIII restriction sites to the 5’ and 3’ ends, respectively, and subsequently cloned into pGL3-basic. Plasmid pRL-TK was obtained from Promega. The Act1-FLAG construct was generated by PCR amplification, using a cDNA clone from GeneCopoeia as the template (cat #FL09699), and cloned into p3XFLAG-CMV-14 (Sigma-Aldrich) as a NotI-ClaI fragment upstream of the 3XFLAG sequence. This construct was used as a template to generate the Act1∆N33-FLAG (minus residues 1–33), Act1∆Bbox-FLAG (minus residues 91–134), Act1∆HLH-FLAG (minus residues 126–180), Act1∆Ubox-FLAG (minus residues 265–328) and Act1∆C401-FLAG (minus residues 401–565) by PCR amplification, then cloned into p3XFLAG-CMV-14 as a NotI-ClaI fragment. For constructs with an internal deletion (Act1∆Bbox-FLAG, Act1∆HLH-FLAG, Act1∆Ubox-FLAG), the N- and C-terminal regions were first generated as NotI-NheI and NheI-ClaI PCR fragments, joined by ligation at the NheI restriction site, then cloned into p3XFLAG-CMV-14. To generate an untagged Act1 expression construct, a stop codon was inserted between the Act1 and 3X-FLAG sequence. Act1-myc/His was generated by PCR amplification of the cDNA clone and cloned into pcDNA3.1-myc/His-A (Invitrogen) as a NotI-XhoI fragment upstream of the tandem myc and 6His tags. Insert identity was confirmed by DNA sequencing for all constructs prior to use. Plasmids were transfected into HBE1 cells using Lipofectamine 2000 (Invitrogen), according to the manufacturer’s suggested protocol.

### 3. Luciferase Assay

HBE1 cells were co-transfected with promoter luciferase constructs, pRL-TK and p3XFLAG-CMV-14 or Act1-FLAG using Lipofectamine 2000, according to the manufacturer’s suggested protocol. 24 hours post-transfection, cells were washed in PBS and lysed in 1X Passive Lysis Buffer, and dual luciferase assay was performed using the Dual-Glo luciferase assay kit (Promega), and luminescence was read using a Lumicount luminometer (Packard Instrument). Firefly luciferase activity was normalized to Renilla luciferase activity to control for variability in transfection efficiency.

### 4. siRNA Knockdown

HBE1 cells (in 24-well plates) were transfected with 6 pmol siRNA using Lipofectamine RNAiMAX (Invitrogen) according to the manufacturer’s suggested protocol. For cells transfected with TRAF6 siRNA, 24 hours post siRNA transfection, cells were either transfected with p3XFLAG-CMV-14 or Act1-FLAG using Lipofectamine 2000. 24h post-transfection, cells were harvested for RNA isolation and real-time PCR analysis, or protein extraction and western blot analysis. For Act1 siRNA transfected cells, cells were treated with IL-17A 48 hours post-transfection for an additional 17 hours.

### 5. RNA Isolation and Real-time PCR

Total RNA was isolated using Nucleospin RNA columns (Machery-Nagel, Düren, Germany) according to the manufacturer’s protocol. cDNA was synthesized from 1 μg total RNA using M-MLV reverse transcriptase (Applied Biosystems Inc) and Oligo(dT) reverse primer. Real-time PCR analysis was performed on an ABI 7900HT PCR system, using Fast-Start SYBR-Green Master Mix (Roche). Real-time primer sequences are listed in Table 1. C(t) values were normalized to a housekeeping gene, *GAPDH*, and calibrated to control group, followed by linearization (2^-∆∆Ct^).

**Table 1:** PCR Primers.

Primer Set	Direction	Sequence (5' --> 3')
CCL20	For	CTGGCTGCTTTGATGTCAGT
	Rev	CGTGTGAAGCCCACAATAAA
DEFB4	For	GCAGGTAACAGGATCGCCTA
	Rev	ATCAGCCATGAGGGTCTTGT
GAPDH	For	CAATGACCCCTTCATTGACC
	Rev	GACAAGCTTCCCGTTCTCAG
IL19	For	CAGGAACAGAGGCAGTGTCA
	Rev	CAGGGATTTAATGGCAGCA
LCN2	For	GGAGCTGACTTCGGAACTAAAGG
	Rev	TGTGGTTTTCAGGGAGGCC
S100A7	For	TGCTGACGATGATGAAGGAG
	Rev	ATGTCTCCCAGCAAGGACAG
S100A8	For	GGGATGACCTGAAGAAATTGCTA
	Rev	TGTTGATATCCAACTCTTTGAACCA
S100A9	For	GTGCGAAAAGATCTGCAAAATTT
	Rev	GGTCCTCCATGATGTGTTCTATGA
S100A12	For	CTGCTTACAAAGGAGCTTGCAA
	Rev	GGCCTTGGAATATTTCATCAATG
DEFB4chIP (-1635 to -1376)	For	CCAGCCCTCTCTTTGCATAC
	Rev	TTACACCTGTCATCCCAGCA
DEFB4chIP (-1508 to -1181)	For	CCATCATGCCTGGCTAATTT
	Rev	TGACAGAGACATGGGGACAA
DEFB4chIP (-1329 to -1118)	For	AAGGATCTTGGCTGCACAAT
	Rev	GGGCAGTTTGAGAAACAAGC
DEFB4chIP (-1163 to -913)	For	ATCACAGACATGGACACTGG
	Rev	TGGGAAACACTTATGAAGGA
DEFB4 chIP (-994 to -786)	For	GGAAGAAGGACAGGGTCCTG
	Rev	GACTTCAGGGCTTTGGGAGC
DEFB4 chIP (-744 to -460)	For	AACTCAAGTGCGGGTGGTAG
	Rev	ATACAGGGCTGGCTCAAACC
DEFB4 chIP (-464 to -254)	For	GTATCATCCCCAGGAGCTGA
	Rev	AACCCAGTGTGTGAATGGAG
DEFB4 chIP (-194 to -20)	For	GGGGTTTCCTGAGTCCAGAT
	Rev	CCACCTTATAAAGGTCCTGG
DEFB4 chIP (-40 to +180)	For	ACCAGGACCTTTATAAGGTGGAAGGC
	Rev	AGGAGACAGAGAACTTCTACGCCA

### 6. Protein Extraction, Co-Immunoprecipitation and Western Blot

For SDS-PAGE analysis, whole cell lysate was prepared by lysing cells in 10 mM Tris-Cl/150 mM NaCl/2 mM EDTA/1% Triton X-100/0.5% sodium deoxycholate + Halt protease inhibitor (Thermo Fisher) for 1 hour at 4°C. Nuclear protein extraction was performed using the PARIS isolation kit (Ambion) according to the manufacturer’s suggested protocol. For co-immunoprecipitation experiments, 1 x 10^7^ cells were lysed in 10 mM Tris-Cl/150 mM NaCl/2 mM EDTA/1% Triton X-100/1X Halt protease inhibitor for 1 hour at 4°C. Cleared lysate was diluted 1:1 in co-immunoprecipitation buffer (10 mM Tris/150 mM NaCl/2 mM EDTA/0.1% Triton X-100/1X Halt protease inhibitor), and incubated with immunoprecipitating antibody overnight at 4°C. 30 μL Protein A/G Plus agarose slurry (Santa Cruz Biotechnology) was added and incubated for an additional hour. The agarose pellets were then washed 3 times in co-immunoprecipitation buffer and boiled in 1X SDS-PAGE loading buffer. Proteins were separated on 4–12% Bis-Tris SDS-PAGE gels (Invitrogen), transferred to PVDF membranes (USA Scientific), blocked in Tris-buffered saline + 0.1% Tween-20 + 5% nonfat dry milk for 1 hour at room temperature and probed with the indicated primary antibodies overnight at 4°C, followed by the appropriate HRP-conjugated secondary antibodies. Before re-probing, blots were stripped with Restore Plus Western Stripping Buffer (Thermo Fisher).

### 7. Chromatin Immunoprecipitation

2.5 x 10^7^ HBE1 cells were transfected with or without Act1-FLAG. 24 hours post-transfection, cells were fixed in 1% paraformaldehyde for 15 minutes, neutralized in 0.125 M glycine, collected and lysed in 100 mM Tris-Cl pH 7.6/10 mM KOAc/15 mM MgOAc/1X Halt protease inhibitor using a 2 mL douncer. Isolated nuclei were then sonicated in 50 mM Tris-Cl, pH 8.0/10 mM EDTA/1% SDS/1X Halt protease inhibitor using a Biorupter (Diagenode), for 30 x 15 second pulses, followed by 1 minute rests to shear chromatin. Sheared chromatin was then precleared, incubated with 1 μg anti-FLAG or control mouse IgG overnight (Abcam) at 4°C, followed by 1 hr incubation with 4 μg rabbit anti mouse IgG (Thermo Fisher), then pulled down with Pansorbin (EMD Biosciences). 10% of the flowthrough from the control mouse IgG immunoprecipitation was kept as “input”. Input and immunoprecipitated material was reverse cross-linked in 0.2M NaCl at 67°C for 18h and analyzed and quantified by PCR. 10% of the sonicated input was used as a positive control for PCR. Non-specific mouse IgG pull-down precipitant was used as a negative control to determine the background noise. The final data was presented as % of input = (intensityAct1 transfectant–mock transfectant) / input *100. For a list of PCR primers and sequences, see Table 1.

### 8. Immunofluorescent Staining

HBE1 cells were plated onto #1.5 glass coverslips (Corning) and allowed to attach overnight. Adherent cells were then transfected with the various Act1 constructs using Lipofectamine 2000. 24h post-transfection, cells were washed in PBS, fixed in 4% paraformaldehyde for 15 minutes, permeabilized in PBS+0.2% Triton-X100 for 5 minutes, blocked for 1 hr in PBS+3% BSA, then incubated overnight at 4°C with 10 ug/mL anti-FLAG (M2) antibody or 200 ng/mL rabbit anti-myc antibody, and 1 hr at room temperature with 1:1000 Alexa-488 conjugated goat anti-mouse secondary antibody. Endogenous Act1 was visualized by incubating with 2 ug/mL anti Act1 antibody and 1:1000 Alexa-488 conjugated goat anti-rabbit secondary antibody. Coverslips were mounted onto Superfrost slides (Thermo Fisher) using Vectashield Mounting Media with DAPI (Vector Labs) and visualized using an LSM710 confocal microscope (Zeiss) outfitted with a 63X oil objective lens (numerical aperture = 1.4). Acquisition software used was Zen 2009 (Zeiss).

## Results

### 1. IL-17A induces the nuclear translocation of Act1

**Fig 1:**
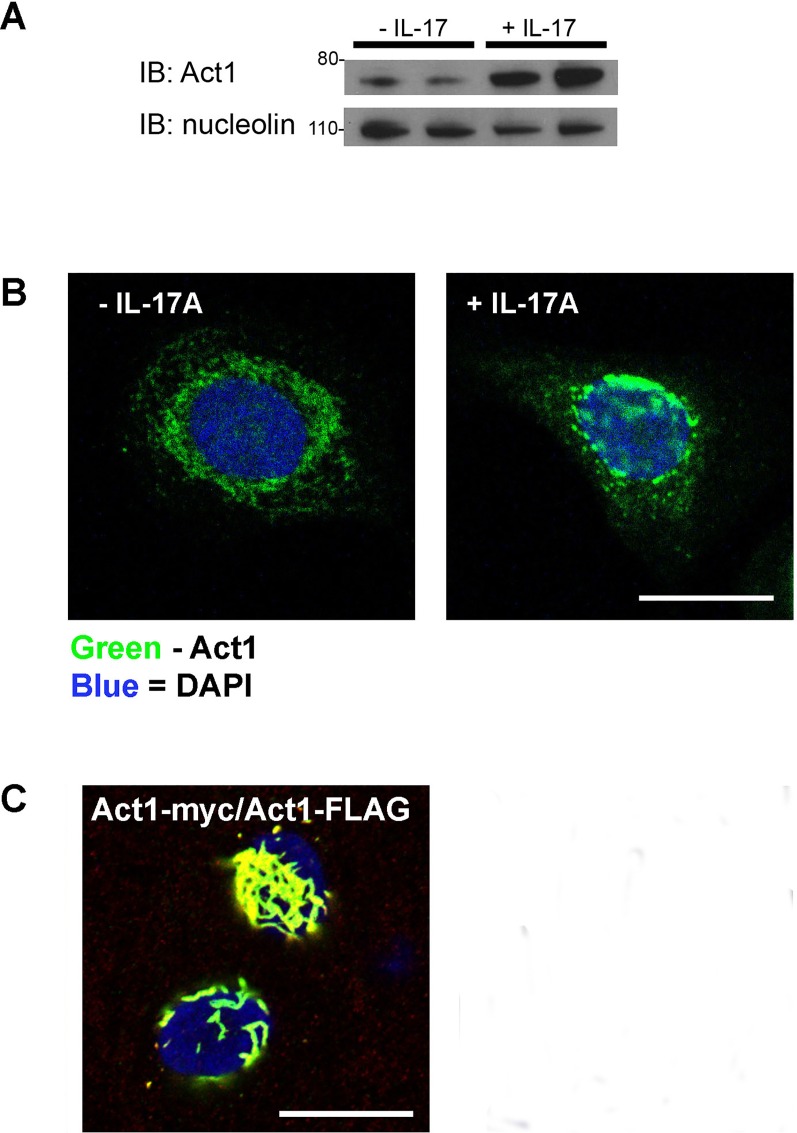
Nuclear localization of Act1. (A) HBE1 were left unstimulated or stimulated with 100 ng/mL IL-17A for 1h prior to nuclear protein extraction. 10 μg nuclear extract was separated by SDS-PAGE and analyzed for Act1 protein expression by Western blot analysis. Nucleolin was used as a protein loading control. (B) HBE1 cells plated on glass coverslips were left unstimulated or stimulated with 100 ng/mL IL-17A for 1h. Endogenous Act1 was immunostained with a rabbit anti-Act1 antibody, followed by an Alexa-488 conjugated secondary antibody (green) and counterstained with DAPI to visualize nuclei (blue). (C) HBE1 cells were transfected with a myc-tagged Act1 expression construct and/or a FLAG-tagged Act1 expression construct. 24 hours post-transfection, cells were fixed and co-stained with rabbit anti myc antibody and mouse anti FLAG antibody, followed by an Alexa-488 conjugated anti-rabbit antibody (green) or Alexa-562 conjugated anti-mouse antibody (red) and counterstained with DAPI to visualize nuclei (blue). Co-localization is indicated in yellow. All confocal images were taken using a 63X objective lens. Bar = 20 μm.

Although Act1 is reported to be a cytoplasmic protein, we found that it is present in the nucleus as well. IL-17A stimulation increased the nuclear localization of endogenous Act1 in an immortalized airway epithelial cell line, HBE1 (Fig 1A). Immunofluorescence analysis confirmed the increased nuclear presence of endogenous Act1 following IL-17A stimulation (Fig 1B). Ectopic expression of Act1 in HBE1 cells resulted in a filamentous staining pattern for Act1 that was concentrated in the nuclear/perinuclear region of the cell. To determine whether the filamentous clustering was due to the ability of Act1 to form oligomers, HBE1 cells were co-transfected with FLAG-tagged Act1 and myc-tagged Act1. Co-staining with anti-FLAG and anti-myc antibodies demonstrated the presence of both proteins within the clusters (Fig 1C). To ensure that the staining pattern was not due to the presence of the C-terminal 3XFLAG tag, HBE1 cells were transfected with an untagged Act1 construct, which resulted in a similar staining pattern (S1 Fig).

### 2. Modulation of Act1 expression levels affects the basal expression of DEFB4

**Fig 2:**
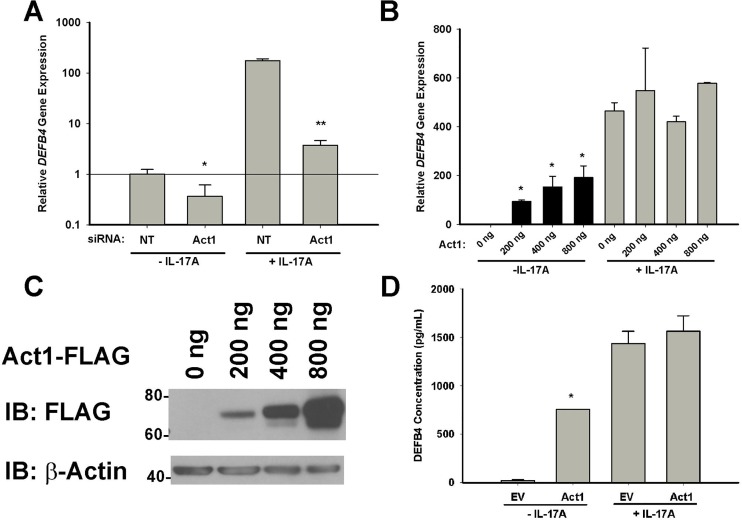
Act1 drives gene expression independent of IL-17 stimulation. (A) HBE1 cells were transfected with Act1 siRNA or a non-targeting (NT) siRNA control. 48h post transfection, cells were treated with media only (-IL-17A) or 100 ng/mL IL-17A (+IL-17A) for an additional 17h, then harvested for RNA extraction and real-time PCR analysis of *DEFB4* expression. C(t) values were normalized to a housekeeping gene, *GAPDH*, and calibrated to NT siRNA/-IL-17A. Error bars represent SEM of 3 independent experiments. *p<0.05 compared to NT siRNA/-IL-17A samples, **p<0.05 compared to NT siRNA/+IL-17A samples. (B) HBE1 cells were transfected with increasing amounts of Act1-FLAG plasmid. Total transfected DNA was normalized using p3XFLAG-CMV-14. 24h post-transfection, cells were treated with media only or 100 ng/mL IL-17A for an additional 17h, then harvested for RNA extraction and real-time PCR analysis of *DEFB4* expression. *p<0.05 compared to 0 ng transfected (mock transfection) (C) To confirm increasing Act1-FLAG protein expression, 30 μg whole cell extracts from transfected cells were analyzed by Western blot for Act1-FLAG. β-actin was used as a loading control. (D) DEFB4 protein was quantitatively measured in cell culture supernatant by ELISA (n = 3). *p<0.05 compared to empty vector (EV) transfected cells.

We used an siRNA approach to knock down the endogenous expression of Act1 in HBE1 cells, prior to IL-17A stimulation. Reducing Act1 expression not only attenuated IL-17A induced *DEFB4* gene expression as previously reported, but attenuated *DEFB4* gene expression in unstimulated cells as well (Fig 2A). Act1 knockdown at the protein level was confirmed by western blot (data not shown). To further characterize the role of Act1 in *DEFB4* expression, HBE1 cells expressing various amounts of Act1-FLAG were treated with or without IL-17A for 17 hours. Act1 expression alone increased *DEFB4* gene expression in unstimulated cells in a dose-dependent manner, but did not significantly increase IL-17A induced *DEFB4* gene expression at any dose (Fig 2B). Increasing amounts of Act1-FLAG protein in whole cell lysates was confirmed by western blot (Fig 2C). ELISA analysis confirmed that increased DEFB4 protein levels were present in the cell culture supernatants of Act1-FLAG transfected cells, compared to empty-vector transfected cells, at the highest dose and that ectopic Act1 expression in combination with exogenous IL-17A stimulation did not further increase DEFB4 protein levels (Fig 2D).

### 3. Further characterization of Act1 induced gene expression

**Fig 3:**
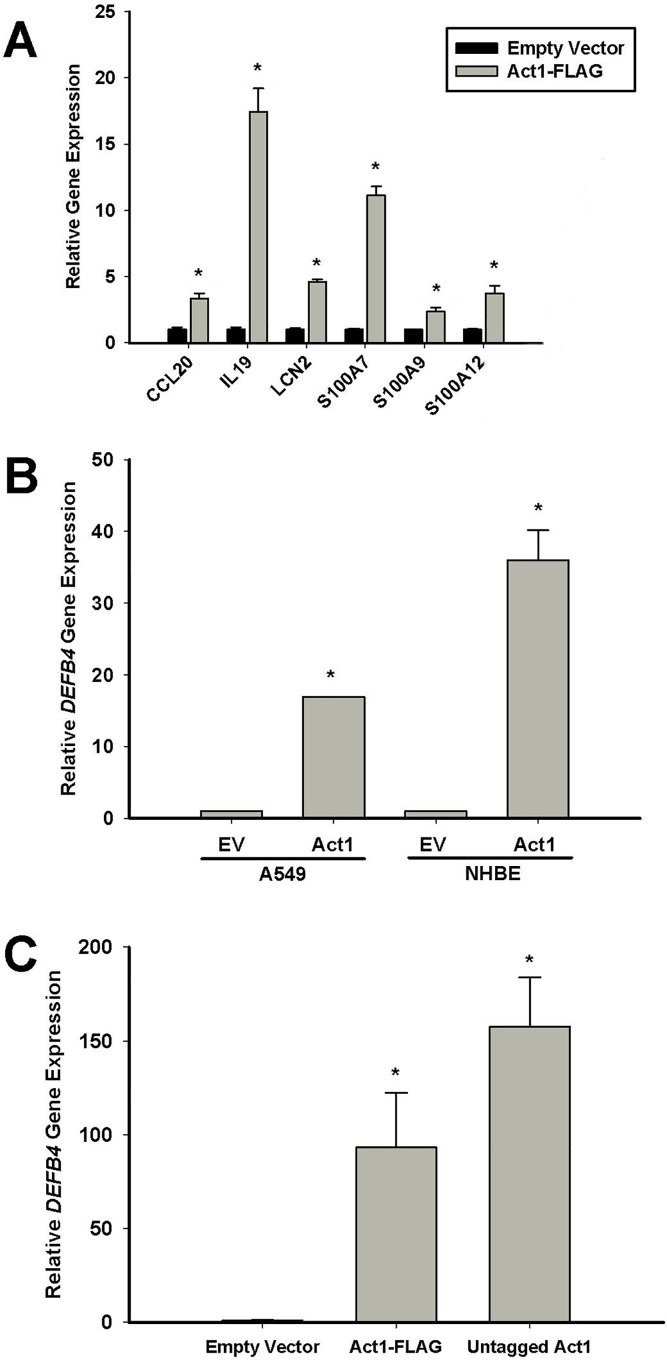
Further characterization of Act1 induced gene expression. (A) HBE1 cells were transfected with Act1-FLAG and harvested 24h post-transfection for RNA extraction and real-time PCR analysis of various IL-17 target genes. (B) A549 or NHBE cells were transfected with empty vector (EV) or Act1-FLAG and harvested 24h post-transfection for RNA extraction and real-time PCR analysis of *DEFB4* expression. (C) HBE1 cells were transfected with empty vector, Act1-FLAG or untagged Act1 and harvested 24h post-transfection for RNA extraction and real-time PCR analysis of *DEFB4* expression. C(t) values were normalized to *GAPDH* and calibrated to empty vector controls. Error bars represent SEM of 3 independent experiments. *p<0.05 compared to empty vector (EV) transfected cells.

In addition to *DEFB4*, we found that a number of previously established IL-17A target genes were upregulated by Act1 expression, including: *CCL20*, *IL-19*, *LCN2*, *S100A7*, *S100A9*, *S100A12* (Fig 3A). We demonstrated that Act1 induced *DEFB4* expression was not exclusive to HBE1 cells, but could also be observed in both primary bronchial epithelial cells, as well as in another lung epithelial cell line, A549 (Fig 3B). Interestingly, we have previously found that *DEFB4* expression is not induced by IL-17A stimulation alone in A549 cells [35]. To demonstrate that the effect on *DEFB4* gene expression was not due to the presence of the C-terminal 3XFLAG tag, HBE1 cells were transfected with a construct containing a non-tagged version of Act1, which yielded similar results (Fig 3C).

### 4. Act1 drives DEFB4 and CCL20 promoter activity

**Fig 4:**
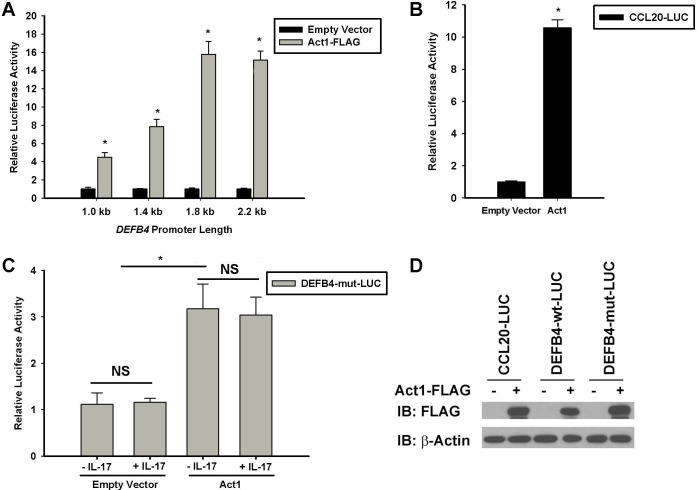
Act1 drives target gene promoter activity. (A) HBE1 cells were transiently co-transfected with *DEFB4* promoter-luciferase constructs, pRL-TK and either empty vector or Act1-FLAG (n = 6) and lysed 24 hours post-transfection. *p<0.05 compared to empty vector (EV) transfected cells. (B) HBE1 cells were co-transfected with a *CCL20* promoter-luciferase construct (CCL20-LUC), pRL-TK and either empty vector or Act1-FLAG (n = 4) and lysed 24 hours post-transfection. *p<0.05 compared to empty vector (EV) transfected cells. (C) HBE1 cells were co-transfected with a *DEFB4* promoter-luciferase construct in which all 3 NF-κB binding sites are mutated (DEFB4-mut-LUC), pRL-TK and either empty vector or Act1-FLAG. 24 hours post-transfection, cells were left unstimulated (-IL-17A) or stimulated with 100 ng/mL IL-17A for 6 hours (n = 4). Lysate was measured for firefly and *Renilla* luciferase activity. Firefly luciferase activity was normalized to *Renilla* luciferase activity and calibrated to empty vector transfected controls. *p<0.05 compared to empty vector (EV) transfected cells. NS: not significant when IL-17A treated cells (+IL-17) were compared to non-treated cells (-IL-17). (D) Lysate was separated by SDS-PAGE and immunoblotted to confirm Act1-FLAG expression. β-actin was used as a loading control.

IL-17A controls gene expression at both the transcriptional and post-transcriptional level [5, 36, 37]. We have previously shown that IL-17A induces *CCL20* and *DEFB4* expression at the transcriptional level [4, 5, 34]. To determine whether or not Act1 promotes *DEFB4* expression at the transcriptional level, HBE1 cells were co-transfected with either empty vector or Act1-FLAG, pRL-TK, and a firefly luciferase reporter construct containing a 1.0 kb, 1.4 kb, 1.8 kb, or 2.2 kb region of the *DEFB4* promoter (Fig 4A). Firefly luciferase readings were normalized to *Renilla* luciferase readings. Act1 increased luciferase activity when compared to empty vector transfected cells for the 1.0 kb– 2.2 kb length *DEFB4* promoter-luciferase reporter constructs, indicating that Act1 drives *DEFB4* expression at the transcriptional level by increasing promoter activity. Act1 was also shown to control *CCL20* expression at the transcriptional level, using a *CCL20* promoter-luciferase construct (Fig 4B). Additionally, Act1 also increased luciferase activity independent of IL-17 treatment for a 2.2 kb *DEFB4* promoter-luciferase reporter construct in which all potential NF-κB binding sites have been mutated (DEFB4-mut-LUC), indicating that the effect is NF-κB independent (Fig 4C). Interestingly, IL-17A stimulation could not increase the luciferase activity of this reporter construct in the absence or presence of Act1, consistent with our previous studies [34]. Act1-FLAG protein expression for all promoter-luciferase studies was confirmed by western blot (Fig 4D).

### 5. Act1 activity is dependent on both the N- and C-terminal regions

**Fig 5:**
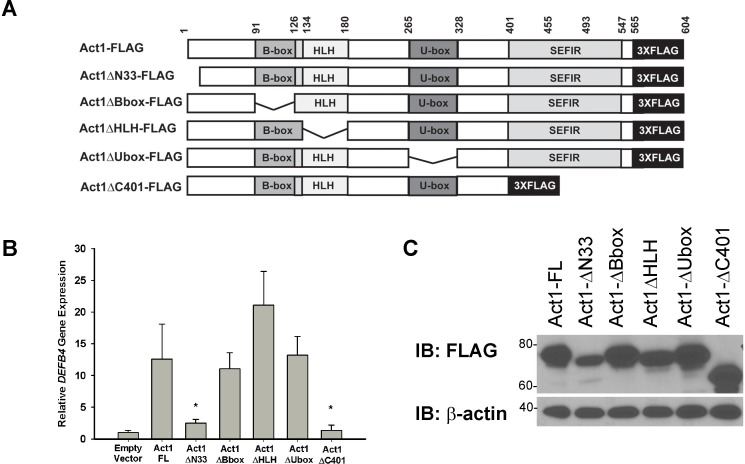
Regions of Act1 necessary for gene expression. (A) Schematic representation of various FLAG-tagged Act1 derivatives. Numbers represent amino acid residue location. (B) HBE1 cells were transiently transfected with empty vector or various Act1 derivatives. 24h post-transfection, cells were lysed for RNA extraction and real-time PCR analysis of *DEFB4* gene expression. C(t) values were normalized to *GAPDH* and calibrated to the empty vector control. Error bars represent SEM of 3 independent experiments. * p< 0.05 compared to Act1-FL (C) 30 μg whole cell lysate was immunoblotted with anti-FLAG antibody to confirm expression of FLAG-tagged Act1 derivative. β-actin was used as a loading control.

To determine which regions of Act1 were necessary for this activity, we created a series of Act1 deletion mutants (Fig 5A). We made deletions of the N-terminal region containing the TRAF6 binding domain (Act1∆N33, minus residues 1–33), the B-box zinc finger domain (Act1∆Bbox, minus residues 91–134), the helix-loop-helix domain (Act1∆HLH, minus residues 126–180), the U-box E3 ubiquitin ligase domain (Act1∆Ubox, minus residues 265–328) or the C-terminal region containing the SEFIR domain (Act1∆C401, minus residues 401–565). All residue notations are based on (shorter) isoform 2 of Act1, which is the major form expressed in the HBE1 and A549 cell lines, as well as in NHBE cells[38]. The B-box zinc finger domain was identified by a SMART database search (http://smart.embl-heidelberg.de); the other domains have been previously described [19, 20, 25, 27]. HBE1 cells were transfected with the various Act1 derivatives and harvested 24 hours post-transfection for RNA isolation and analyzed for *DEFB4* gene expression. Deletion of the B-box zinc finger, helix-loop-helix, or U-box E3 ubiquitin ligase domains had no detrimental effect on *DEFB4* expression when compared to full-length Act1 transfected cells. In comparison, deletion of either the N- and C-terminal regions significantly attenuated Act1’s ability to induce *DEFB4* expression (Fig 5B). Protein expression of the Act1 derivatives was confirmed by western blot (Fig 5C).

### 6. Act1 binds to the promoter of DEFB4

**Fig 6:**
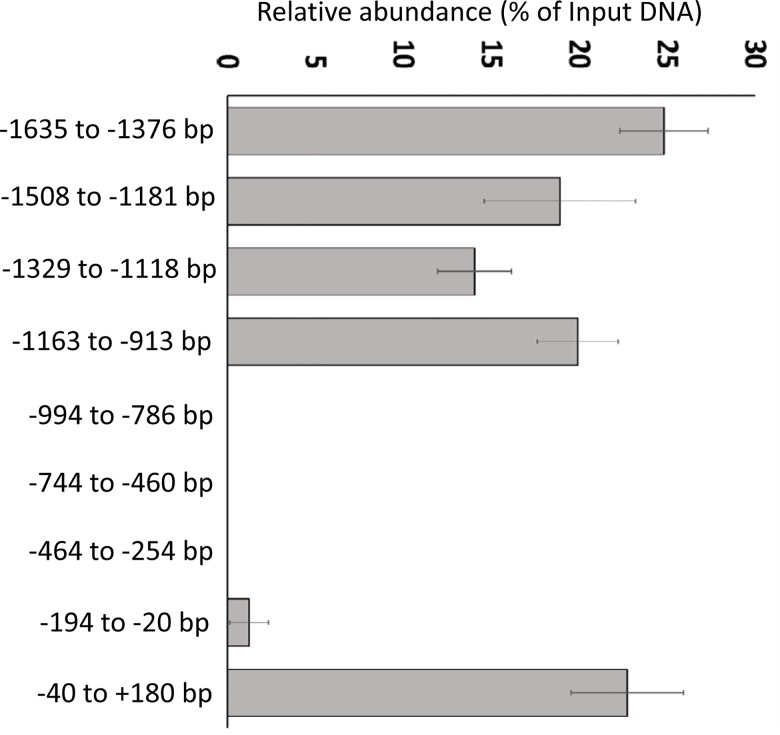
Nuclear Act1 binds to the DEFB4 promoter regions. HBE1 cells were transfected with Act1-FLAG and fixed 24h post-transfection. Isolated nuclei were sonicated and chromatin immunoprecipitation (chIP) was performed using an anti-FLAG antibody or non-specific mouse IgG. PCR was used to detect *DEFB4* promoters in the immunoprecipitated material. The data was presented as % of the input as described in Materials and Methods.

Because of its presence in the nucleus, we sought to determine if nuclear Act1 could bind to the promoter of its target genes. Chromatin immunoprecipitation (chIP) was performed on cells ectopically expressing Act1, followed by PCR analysis. We found that Act1 was able to bind to the *DEFB4* promoter in the region -1635 bp to -913 bp upstream of the transcriptional start site, and also at the transcriptional start site (-40 bp to +180 bp (Fig 6).

## Discussion

In this study we demonstrate for the first time that Act1 is present in the nuclear compartment of airway epithelial cells and can function as a transcriptional enhancer. Western blot and immunofluorescence analyses of Act1 revealed nuclear localization of both endogenously and ectopically expressed full-length Act1. Nuclear localization of endogenous Act1 was enhanced with IL-17A stimulation, indicating a possible role for nuclear Act1 in IL-17A signaling. How Act1 enters the nucleus is still unclear; Act1 does not contain any canonical nuclear localization signals, but may enter the nucleus through another method. Previously, Act1 has been described as a cytoplasmic adaptor or scaffolding protein that binds to the IL-17 receptor and activates the downstream NF-κB pathway by associating with other NF-κB pathway proteins such as IKK-α/β, TAK1 and TRAF6. Our data suggests that Act1 may have dual functions; as an adaptor protein and E3 ubiquitin ligase in the cytoplasm, and as a transcriptional enhancer in the nucleus. Other membrane-associated scaffolding proteins that have been shown to also have a nuclear function include zonula occluden (ZO) proteins and X11/MINT family members [39, 40].

We demonstrated that Act1 could up-regulate a subset of IL-17A target genes, including *DEFB4*, *IL-19 and CCL20* by binding to and directly inducing promoter activity. The effect of Act1 on gene expression is dependent on both its N- and C-terminal regions. The N-terminal region encompasses the newly identified TRAF6 binding domain, and the C-terminal region encompasses the SEFIR domain, but the exact role of these domains has yet to be determined. Previous studies have shown that these domains are necessary for NF-κB activation [20, 29]. Although we were able to demonstrate that full-length Act1 could increase NF-κB activity, similar to previously reported studies [19, 20, data not shown], the effect on transcriptional activity was NF-κB independent, as Act1 was able to up-regulate gene expression in the presence of an NF-κB inhibitor, and also able to activate a *DEFB4* promoter reporter construct in which all NF-κB sites have been mutated. Additionally, deletion of the E3 ubiquitin ligase domain, which has been shown to be necessary for IL-17 mediated NF-κB activation [27], had no effect on Act1-mediated gene expression.

Using chromatin immunoprecipitation, we showed that Act1 binds to the promoter of *DEFB4* in the region of -1635 bp to -913 bp upstream of the transcriptional start site, as well as at the transcriptional start site, which correlates to our reporter assay data. It is not clear why Act1 binds to such a large region of DNA, or whether or not Act1 binds DNA directly as a transcription factor, or indirectly as co-activator via another as yet unidentified DNA binding protein. Analysis of various Act1 deletion mutants indicates that the latter is likely the case, as deletion of potential DNA binding motifs, such as the zinc finger B-box domain and the helix-loop-helix motif, had no effect on Act1 induced gene expression. It is possible that nuclear Act1 functions as a bridging protein that modulates the binding of other transcription factors to the promoter of certain target genes. Although we demonstrate that IL-17A stimulation induces Act1 nuclear localization, it is unclear whether it also induces its DNA binding. Technical difficulties prevented us from examining the DNA binding activity of endogenous Act1, possibly because of difficulties in immuno-precipitating Act1 from paraformaldehyde fixed cells, and the considerably smaller fraction of endogenous Act1 that is in the nucleus. Global transcriptome analysis techniques such as oligonucleotide microarray, RNAseq or chIP-chip will be needed to identify other classes of genes regulated by Act1. In particular, it will be interesting to see if IL-25 and CD40 target genes are affected, as epithelial Act1 has been shown to play a critical role in mediating IL-25 signaling in the airway and a negative regulatory role for CD40 signaling [17, 41].

In this study, we have described a unique transcriptional regulatory function for the critical IL-17A signaling component, Act1. In airway epithelial cells, Act1 controls the expression of several key antimicrobial proteins and cytokines that play a role in both the innate immune response and the generation of an adaptive immune response. These results support the notion that while cytoplasmic Act1/IL-17 receptor interaction is responsible for IL-17 mediated canonical NF-kB activation and while the majority of IL-17 mediated gene expression, nuclear Act1 alone serves as a transcriptional activator to up-regulate basal gene expression in a novel, NF-kB independent manner. In the presence of canonical signaling, this pathway cannot further stimulate IL-17 induced gene expression. The mechanisms by which these two pathways are controlled deserve further characterization and delineation to understand the dual role of intracellular Act1.

## Supporting Information

S1 Fig HBE1 cells were transfected with an untagged Act1 construct, fixed 24 hours post-transfection and stained with anti-Act1 antibody (green) and counter-stained with DAPI (blue). Magnification = 60X. Bar = 20um
(TIF)
