# Supplementary figures and images for: A Novel Nuclear Function for the Interleukin-17 Signaling Adaptor Protein Act1
Source: PLoS One. 2016 Oct 10;11(10):e0163323. doi: 10.1371/journal.pone.0163323 (PMC5056742; doi:10.1371/journal.pone.0163323)

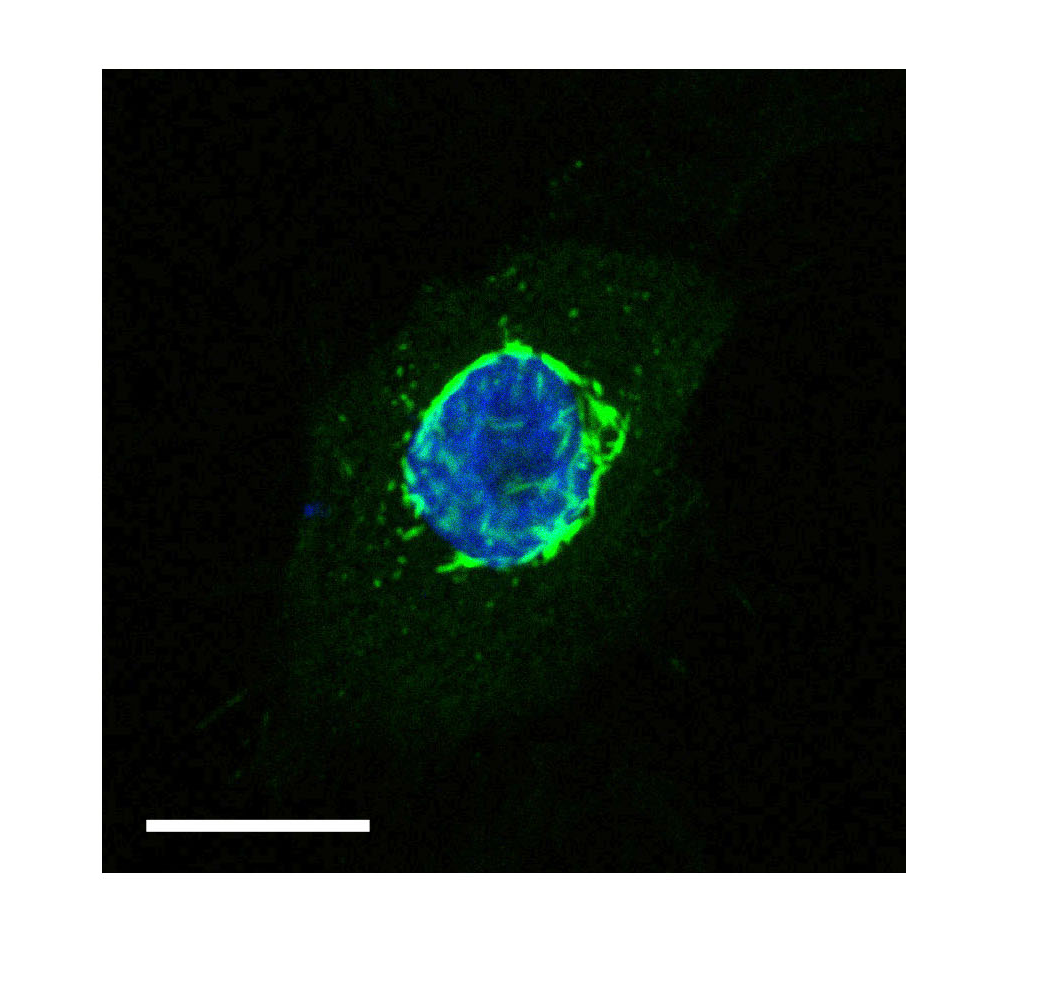

Supplement: S1 Fig — Magnification = 60X. Bar = 20um (TIF) [file pone.0163323.s001.tif]
